# Prediction of refraction error after toric lens implantation with biometric input data uncertainties and power labelling tolerances

**DOI:** 10.1111/ceo.14449

**Published:** 2024-10-09

**Authors:** Achim Langenbucher, Nóra Szentmáry, Alan Cayless, David Cooke, Peter Hoffmann, Jascha Wendelstein

**Affiliations:** ^1^ Department of Experimental Ophthalmology Saarland University Saarbrücken Germany; ^2^ Dr. Rolf M. Schwiete Center for Limbal Stem Cell and Aniridia Research Saarland University Saarbrücken Germany; ^3^ Department of Ophthalmology Semmelweis‐University Budapest Hungary; ^4^ School of Physical Sciences The Open University Milton Keynes UK; ^5^ Great Lakes Eye Care Saint Joseph Michigan USA; ^6^ Department of Neurology and Ophthalmology, College of Osteopathic Medicine Michigan State University East Lansing Michigan USA; ^7^ Augen‐ und Laserklinik Castrop‐Rauxel Castrop‐Rauxel Germany; ^8^ Department of Ophthalmology Johannes Kepler University Linz Linz Austria

**Keywords:** biometric measure uncertainties, lens power labelling tolerances, Monte‐Carlo simulation, refractive outcome prediction, toric lenses

## Abstract

**Background:**

The purpose of this study was to simulate the impact of biometric measure uncertainties, lens equivalent and toric power labelling tolerances and axis alignment errors on the refractive outcome after cataract surgery with toric lens implantation.

**Methods:**

In this retrospective non‐randomised cross sectional Monte‐Carlo simulation study we evaluated a dataset containing 7458 LenStar 900 preoperative biometric measurements. The biometric uncertainties from literature, lens power labelling according to ISO 11979, and axis alignment tolerances of a modern toric lens (Hoya Vivinex) were taken to be normally distributed and used in a Monte‐Carlo simulation with 100 000 samples per eye. The target variable was the defocus equivalent (DEQ) derived using the Castrop (DEQ_C_) and the Haigis (DEQ_H_) formulae.

**Results:**

Mean/median / 90% quantile DEQ_C_ was 0.22/0.21/0.36 D and DEQ_H_ was 0.20/0.19/0.32 D. Ignoring the variation in lens power labelling and toric axis alignment the respective DEQ_C_ was 0.20/0.19/0.32 D and DEQ_H_ was 0.18/0.17/0.29 D. DEQ_C_ and DEQ_H_ increased with shorter eyes, steeper corneas, equivalent lens power and highly with toric lens power.

**Conclusions:**

According to our simulation results, uncertainties in biometric measures, lens power labelling tolerances, and axis alignment errors are responsible for a significant part of the refraction prediction error after cataract surgery with toric lens implantation. Additional labelling of the exact equivalent and toric power on the lens package could be a step to improve postoperative results.

## 1 INTRODUCTION

There are several reasons for refractive surprises after cataract surgery in terms of deviation of the achieved refraction from the target refraction^1^, ^2^, ^3^, ^4^, ^5^, ^6^, ^7^: (A) the recorded biometric parameters used for the lens power calculation may be inappropriate or inaccurate,^8^, ^9^, ^10^, ^11^ (B) the labelled power of the intraocular lens may not properly describe the refraction of the lens in the eye,^4^, ^7^ (C) the lens power calculation strategy may be inappropriate,^6^, ^7^, ^12^, ^13^ and (D) there may be deterministic or stochastic changes in the biometric measures due to surgery.^1^, ^6^ With (A) there might be systematic errors for example, from incorrect calibration of the biometric device, model assumptions / simplifications which are not valid for the patient eye or simply measurement noise in terms of technical variability of the biometric parameters.^8^, ^9^, ^10^, ^11^ With (B) ISO standard EN ISO 11979‐2:2014^14^ gives us guidelines on the measurement techniques for intraocular lenses (IOL) and strict benchmarks on the labelling of the equivalent or toric power.^15^ With (C) the assumptions made in the lens power formula or the simplifications of the model could be invalid or the (optimised) formula constants could be inappropriate.^6^, ^7^, ^16^ With (D) the most common effects are that the geometry of the cornea could change (e.g. flattening or steepening of the cornea) or some corneal astigmatism could be induced by the corneal incision.^6^, ^16^

Even though biometers have improved significantly over the last two decades, with modern optical biometers having excellent repeatability,^7^ all biometric measures will still be subject to some amount of noise and this has to be taken into account in the lens power calculation strategy. There are many papers on the repeatability or reproducibility of biometers, but most of them are based on multiple measurements in a single session (intrasession repeatability) and this limitation might underestimate the true variability of the biometric measures.^8^, ^9^, ^10^, ^11^

There is also some noise in the labelled lens power.^4^, ^5^, ^15^ For stigmatic lenses we have to consider only the variation in the IOL equivalent power. According to ISO 11979 the labelling tolerance increases with the lens power from ±0.3 dioptres (D) for low power IOLs (0–15 D) to ±1.0 D for high power IOLs (more than 30 D).^14^ For toric lenses, in addition to the tolerance in the labelling of the equivalent lens power we have to consider the variations in the toric power and in the alignment of the marked toric lens axis with the steep corneal meridian. As with the IOL equivalent power, the labelling tolerance for the toric lens power allowed by the standard increases in stages, starting at ±0.3 D for low lens toricities (up to 2.5 D) and low overall power, increasing to ±0.4 D for overall equivalent powers greater than 25 D, and increasing again to ±0.5 D for IOL toricities greater than 4.5 D.^14^ The variability in the alignment of the toric axis is well documented, having been extensively studied by several researcher groups in the past.^8^, ^9^, ^10^, ^11^

All of these uncertainties have the potential to affect the refractive outcome after cataract surgery with IOL implantation.^5^, ^12^, ^13^, ^17^ For stigmatic IOLs we typically consider the deviation of the spherical equivalent of the achieved postoperative refraction from the target refraction.^1^, ^7^, ^12^, ^13^ However, for toric IOLs the prediction error in spherical equivalent is not sufficient, and we have instead to consider the defocus equivalent. This describes the length of the 3D refraction power vector and takes into account the deviations from target refraction in both spherical equivalent and refractive cylinder.

The purpose of this study was to use literature data for the measurement noise of biometric parameters, the labelling tolerances for the equivalent and toric lens power according to ISO 11979, and the literature data for the alignment error for the axis of toric lenses to set up a Monte‐Carlo simulation:

1. to investigate the predicted defocus equivalent error after cataract surgery with toric lens implantation
2. to evaluate the predicted defocus equivalent error as a function of the biometric measures and the power of the implanted lens
3. to study the contributions of the biometric parameters and the lens power and axis alignment on the predicted defocus equivalent error

## 2 METHODS

### 2.1 Dataset for the prediction model

A large dataset containing 12 703 biometric measurements was considered in this study. All measurements were performed at the Augen‐ und Laserklinik, Castrop‐Rauxel, Germany with the LenStar900 (Haag‐Streit, Köniz, Switzerland). The Institutional Review Board provided a waiver for this study (Ärztekammer des Saarlandes, 157/21). Informed consent of the patients was not required. The study followed the tenets of the Declaration of Helsinki.

The data were anonymised at source and transferred to a .csv data table using the software module for batch data export. Data tables were reduced to the relevant parameters required for our data analysis, consisting of the following measurements: from the measurement before cataract surgery we extracted the date of birth and the measurement date, the laterality (left or right eye), gender (female or male), flat (*R*1_a_) and steep (*R*2_a_) corneal front surface radii of curvature both in mm, axis of the flat meridian (Ax) in degree, axial length (AL) in mm, central corneal thickness (CCT) in mm, anterior chamber depth (ACD) in mm (measured from corneal epithelium to lens), and central thickness of the crystalline lens (LT) in mm. Subjects with missing data or data outside our simulation parameter space (AL: 18–34 mm; CCT: 380–750 μm; ACD: 1.8–4.3 mm; LT: 3.0–5.5 mm; *R*1_a_ and *R*2_a_: 6.5–9.5 mm) were excluded. The data were transferred to Matlab (MathWorks, Natick, USA) for further processing.

### 2.2 Data pre‐processing in Matlab

The patient age in years was calculated from the time interval between date of birth and examination date before cataract surgery. The mean corneal curvature *R*_a_ in mm was derived as the harmonic mean from the corneal curvature in the flat and steep meridians as *R*_a_ = 0.5·*R*1_a_·*R*2_a_/(*R*1_a_ + *R*2_a_). The mean keratometric power was calculated as $\frac{168.75}{R1_{a}}+\frac{168.75}{R2_{a}}$ and keratometric astigmatism was calculated as $\frac{337.5}{R2_{a}}-\frac{337.5}{R1_{a}}.$ For the intraocular lens power calculation we implemented the Haigis formula^18^ as an example of a fully disclosed 4th generation lens power calculation concept, and the Castrop formula^19^, ^20^, ^21^ as a modern lens power calculation formula dealing with a thick lens model for the cornea and an effective lens position prediction which resamples the anatomically correct axial position of the IOL in the pseudophakic eye. To simplify the data interpretation, instead of the measured corneal back surface data we used a corneal back surface derived from a fixed front to back surface ratio (7.77 mm/6.4 mm) according to the schematic model eye of Liou & Brennan.^22^ As examples we considered for our calculations a modern widely used model of toric intraocular lens (Vivinex toric XY1A, Hoya Surgical, Singapore). The respective formula constants for the Haigis formula (*a*0/*a*1/*a*2 = −1.0459/0.2547/0.2391) and for the Castrop formula (C/H/R = 0.3249/0.1267/0.1548) were extracted from the IOLCon WEB platform (https://IOLCon.org, accessed on 07 May 2024). The ‘perfect’ or ‘exact’ lens power (equivalent power IOLPEQ in dioptres, toric power IOLPC in dioptres, and target axis IOLPA in degrees) was calculated for each eye using both lens power calculation formulae for a target refraction of −0.1 D (according to the practice of most surgeons to aim for mild myopia).^6^, ^16^ In addition to the IOLCon optimised formula constants we also extracted the lens delivery range for the Vivinex lens from the IOLCon WEB platform (6–30 D in steps of 0.5 D for the equivalent and 1–6 D for the toricity). Eyes with equivalent lens power outside the range 5.75–30.25 D or toric lens power outside the range 0.57.0 D were removed from the dataset.

### 2.3 Monte‐Carlo simulation in Matlab

A Monte‐Carlo simulation is a computational method involving a random element, commonly used to simulate or derive distributions of variables which are subject to intrinsic variation. In a typical Monte‐Carlo simulation, a calculation is repeated a large number of times with random variations applied to the input variables for each repeat. This process generates a predicted, or simulated, distribution of the output parameters. For this study, the first step in generating these variations in the input parameters was to select the best fit lens for each eye by mapping the equivalent power to the closest available IOL equivalent power step (quantised power IOLPEQQ). To avoid overcorrection of corneal astigmatism the continuous lens torus values were quantised into discrete steps IOLPCQ (IOLPC from 0.5 to 1.4 D: IOLPCQ → 1.0 D; 1.4 to 2.15 D: IOLPCQ → 1.5 D; 2.15–2.9 D: IOLPCQ → 2.25 D; 2.9–3.65 D: IOLPCQ → 3.0 D; 3.65–4.4 D: IOLPCQ → 3.75 D; 4.4–5.15 D: IOLPCQ → 4.5 D; 5.15–5.9 D: IOLPCQ → 5.25 D; and 5.9–7.0 D: IOLPCQ → 6.0). For each available power step (IOLPEQQ and IOLPCQ), the labelling tolerance of the selected lens was determined by reference to the ISO Standard (EN ISO 11979‐2:2014),^14^ which specifies the allowed tolerances dependent on the overall equivalent and toric powers. According to Norrby et al.^5^ we used a variation with a standard deviation (Sw) of 1/3 of the permitted ISO tolerances for the labelling of both the equivalent and the toric power. Because the Gaussian distribution for the lens power labelling tolerances as used by Norrby et al. could result in equivalent and toric lens power values outside the ISO limits, we instead implemented a truncated Gaussian distribution, limiting variations to within the ISO labelling tolerances. For the variations of the biometric measures we used the literature data^8^, ^9^, ^11^ of standard deviation for repeat measurements (Sw): AL: 0.017 mm, CCT: 3 μm, ACD: 0.010 mm, LT: 0.016 mm, Ax: 4.7°. The literature data for the variation of the keratometric power in the flat (0.145 D) and steep meridians (0.139 D) as derived with a keratometer index of nK = 1.3375 were individually converted to variations in *R*1_a_ and *R*2_a_.^9^, ^11^ For the corneal back surface radius we assumed a variation proportional to the variation of the corneal front surface radius in the respective meridian. For the alignment of the toric lens axis to IOLPA (the steep corneal meridian) we assumed a variation (Sw) of 3° as derived from a screening of data from several literature sources.^6^

The next step involved setting up a Monte Carlo simulation using the biometric data from the dataset with the variations described above for AL, CCT, ACD, LT, *R*1_a_, *R*2_a_, Ax, the equivalent and toric lens power with variations according to the ISO standards, and the targeted implantation axis of the lens with the variation of the axis.^1^, ^12^, ^13^, ^23^ For all parameters, in the absence of more detailed information on the error distribution in the literature^12^, ^13^ we assumed normally distributed and uncorrelated parameter uncertainties.^12^, ^13^ For each eye and for both formulae, NMC = 100 000 Monte‐Carlo samples were traced. From the predicted spherocylindrical refraction (spherical equivalent SEQ and refractive cylinder Cyl, both in dioptres) at the spectacle plane, we calculated the defocus equivalent $\mathrm{DEQ}=\sqrt{{\mathrm{SEQ}}^{2}+\frac{1}{4}∙{\mathrm{Cyl}}^{2}}$ as the quality metric. The DEQ was chosen in preference to the SEQ because it best describes the residual spherocylindrical refraction error after toric lens implantation in terms of a single, positive number.^24^, ^25^ In contrast to the SEQ, which quantifies only an ‘average refraction error’, the DEQ combines the deterioration in uncorrected vision resulting from both the spherical equivalent error and the cylindrical error. The DEQ is therefore commonly used in refractive surgery^26^, ^27^ instead of the spherical equivalent because it correlates well with the loss in visual acuity (LogMAR units) resulting from classical defocus or cylindrical refraction errors in the absence of physiological accommodation.^24^, ^25^ In addition, to isolate the effect of variations in the biometric measures, we performed a separate Monte‐Carlo simulation (again with NMC = 100 000 samples per eye) considering the variation in all biometric parameters AL, ACD (for the Haigis and the Castrop formula), CCT and LT (only for the Castrop formula) and in keratometry (*R*1_a_, *R*2_a_ and Ax) but without variations in IOL equivalent and toric lens power or alignment of the toric lens axis resulting from labelling tolerances. Further, to evaluate the impact of variations of each individual predictor, we performed separate Monte‐Carlo simulations considering variations in just one parameter at a time. These covered variations in AL, ACD (for the Haigis and the Castrop formula), CCT and LT (only for the Castrop formula), keratometry (R1_a_, R2_a_ and Ax), IOL labelling power (equivalent and toric lens power) and alignment of the toric lens axis, with the variations of all other predictors zeroed in each case.

### 2.4 Statistical evaluation

The explorative data analysis presented in the tables was carried out in terms of the arithmetic mean, the standard deviation, the median, and the lower and upper boundaries of the 95% confidence interval (corresponding to the 2.5% and 97.5% quantiles of the distributions). Because the DEQ used as our quality metric is a strictly one‐sided distribution, the arithmetic mean might not be fully representative.^23^ For this reason we also extracted the median and the 90% quantile of the distribution from the 100 000 DEQ samples for each eye. The overall distributions of these DEQ results for the *n* = 7458 eyes in the study are presented as Cumulative Distribution Function (CDF) plots for both formulae under test. These are used to show the contribution or weighting of results in each part of the DEQ range.

The CDF is the integral of the probability density function and represents the probability that an individual sample selected randomly from the population would have a DEQ equal to or less than the corresponding DEQ value at that point.

For a more detailed analysis the mean, median and 90% quantiles of the DEQ were also plotted as scattergraphs as a function of the main input parameters AL, R_a_, equivalent and toric lens power together with the linear best fit lines to better understand the trends for each of these parameters.

## 3 RESULTS

From the *N* = 12 703 measurements in the database transferred to us, *N* = 10 944 fulfilled the initial selection criteria, and after further selection of cases within the lens delivery range a dataset with measurements for *N* = 7458 eyes was used for our analysis. In total, 3696 right and 3762 left eyes were included. Table 1 lists the descriptive data for the ocular biometry for our dataset including age, AL, CCT, ACD, LT, *R*1_a_ and *R*2_a_, *R*_a_, mean keratometric power and keratometric astigmatism. Since, for simplicity, we used a fixed corneal front to back surface curvature ratio for calculating the intraocular lens power with the Castrop formula,^19^, ^20^ the corneal back surface radius data are not listed.

**TABLE 1:** Explorative data of the input parameters used for toric lens power calculation in terms of mean value, standard deviation, median, and lower and upper boundary of the 95% confidence interval (2.5% and 97.5% quantiles).

*N* = 7458	Age in years	AL in mm	CCT in μm	ACD in mm	LT in mm	*R*1_a_ in mm	*R*2_a_ in mm	*R*_a_ in mm	*K*_MEAN_	*K*_AST_ in D
Mean	76.2563	23.825	552	3.2647	4.4341	7.8391	7.61057	7.7224	43.7608	1.2939
Standard deviation	11.2700	1.6859	37	0.3830	0.3590	0.2945	0.2836	0.2790	1.5732	0.8427
Median	77.9042	23.5300	551	3.2650	4.4800	7.8250	7.6100	7.7146	43.7482	1.0358
2.5% quantile	52.5685	21.0795	48	2.4880	3.6200	7.3000	7.0800	7.2034	40.6434	0.5221
97.5% quantile	94.7817	27.9005	62	4.0060	4.9700	8.4500	8.1900	8.3039	46.8526	3.6344

*Note*: Age refers to the patient age at the time of the biometric measurement before cataract surgery, AL to the axial length of the eye, CCT to the central corneal thickness, ACD to the phakic anterior chamber depth as the distance between the corneal epithelium and the front lens apex, LT to the central thickness of the crystalline lens, *R*1_a_ and *R*2_a_ to the corneal radii of curvature in the flat and steep meridians, *R*_a_ to the harmonic mean corneal radius, *K*_MEAN_ to the mean keratometric power, and *K*_AST_ to the keratometric astigmatism.

The equivalent / toric power of the lens according to the Castrop formula^19^, ^20^, ^21^ was calculated as IOLPEQ = 20.49 ± 5.10 D (median 21.20 D, 95% confidence interval from 8.81 to 30.07 D)/IOLPC = 1.79 ± 1.16 D (median1.43 D, 95% confidence interval from 0.86 to 5.04 D). According to the Haigis formula^18^ the respective lens powers were calculated as IOLPEQ = 20.42 ± 5.20 D (median 21.13 D, 95% confidence interval from 8.31 to 29.99 D)/IOLPC = 1.84 ± 1.19 D (median 1.48 D, 95% confidence interval from 0.87 to 5.20 D).

**FIGURE 1:**
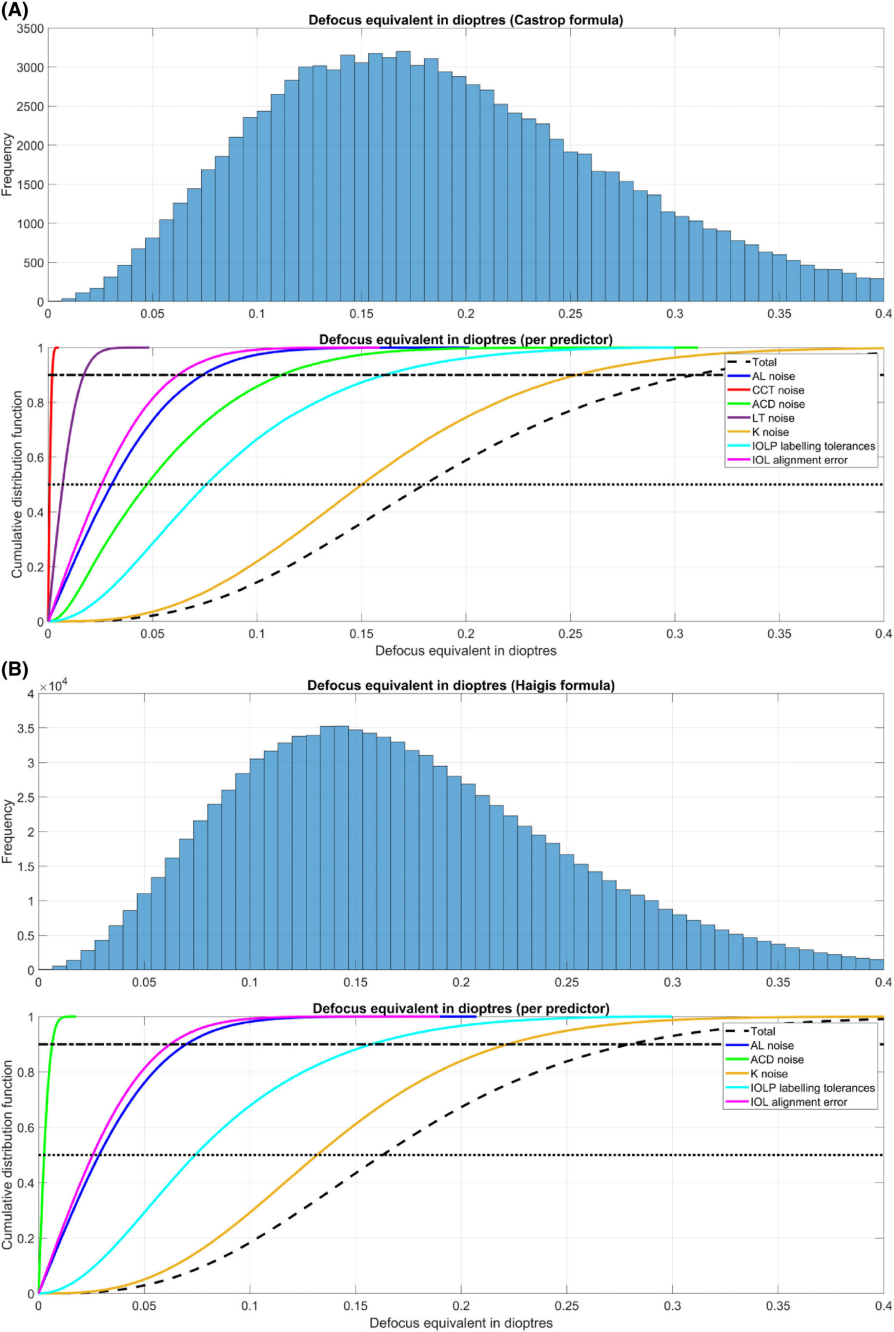
Result of the Monte‐Carlo simulation (100 000 samples) for clinical case #3 (out of 7458 cases) in the dataset. (A) The calculation results with the Castrop formula as an example of a modern lens calculation concept with a thick lens model for the cornea and (B) the corresponding results with the Haigis formula as an example of a classical 4th generation formula. In the upper plot of both graphs the histogram distribution of the predicted defocus equivalent is shown. In the lower plot of both graphs the cumulative distribution functions are displayed for the defocus equivalent for variations in the individual parameters axial length (AL noise), anterior chamber depth (ACD noise), central corneal thickness (CCT noise) and lens thickness (LT noise) (both only shown for the Castrop formula), variation in keratometry (K noise, flat and steep corneal meridian and keratometer axis), variation in lens power labelling (equivalent and toric lens power labelling tolerances), and the variation in the alignment of the axis of the toric lens (IOL alignment error). The dashed black line displays the cumulative distribution function when variations for all input parameters are considered together (Total). In the lower plots, the dotted / dash‐dotted black lines refer to the median / 90% quantile of the distributions respectively.

To give some insight into the strategy of the Monte‐Carlo simulation, we selected case #3 out of *N* = 7458 as an example. This example record has values of: AL = 23.26 mm, CCT = 557 μm, ACD = 3.14 mm, LT = 4.47 mm, *R*1_a_ = 7.92 mm at Ax = 94.72°, *R*2_a_ = 7.78 mm. The lens power according to the Castrop formula was IOLPEQ = 23.25 D/IOLPC = 1.01 D. Considering the respective uncertainties (Sw) in the biometric parameters for AL (17 μm), CCT (3 μm), ACD (10 μm), LT (16 μm), *R*1_a_ (27 μm), *R*2_a_ (25 μm), Ax (4.7°), in the lens power label with IOLPEQ (0.13 D) and IOLPC (0.10 D), and in the toric axis alignment (3°) the Monte‐Carlo simulation with NMC = 100 000 samples yields a histogram distribution for the defocus equivalent DEQ as shown in the upper plot of Figure 1A. The corresponding cumulative distribution function plot is displayed in the lower plot (dashed black line). To show the impact of each individual predictor on the DEQ, the Monte‐Carlo simulation was repeated allowing variation of only one parameter at a time and setting all other uncertainties to zero. Results for each predictor are shown in the lower graph of Figure 1A colour coded as follows: AL (blue line), CCT (red line), ACD (green line), LT (purple line), keratometry (variations in *R*1_a_, *R*2_a_ and Ax, ochre line), lens power labelling (variations in IOLPEQ and IOLPC, cyan line), and the toric axis alignment (magenta line).

The lens power according to the Haigis formula was IOLPEQ = 23.21 D/IOLPC = 1.03 D. Considering the previous uncertainties in the biometric parameters for AL, ACD, *R*1_a_, *R*2_a_, Ax, in the lens power label and in the toric axis alignment (in this case identical to the Castrop formula) the Monte‐Carlo simulation yields a histogram distribution for the defocus equivalent DEQ as shown in the upper plot of Figure 1B. The corresponding cumulative distribution function plot is provided in the lower plot (dashed black line). To show the impact of each individual predictor on the DEQ, the Monte‐Carlo simulation was repeated allowing variation of only one parameter at a time and setting all other uncertainties to zero. Results for each predictor are shown in the lower graph of Figure 1B colour coded as follows: AL (blue line), ACD (green line), keratometry (variations in *R*1_a_, *R*2_a_ and Ax, ochre line), lens power labelling (variations in equivalent and toric lens power, cyan line), and the lens axis alignment with respect to the target axis (magenta line).

Table 2 summarises the descriptive data for the mean, median, and 90% quantile DEQ when the uncertainties in all input parameters are considered (left side) or only the biometric parameter uncertainties are considered (right side) and a toric lens with the exact equivalent and toric lens power is considered exactly at the target axis. In the upper / lower part of the table, the situation is displayed for the Castrop / Haigis formula respectively.

**TABLE 2:** Explorative data of the predicted ‘overall’ defocus equivalent at the spectacle plane resulting from a Monte‐Carlo simulation with 100 000 samples for each eye.

		With IOL labelling and alignment error			Without IOL labelling and alignment error		
DEQ in dioptres; *N* = 7458		Mean	Median	90% quantile	Mean	Median	90% quantile
Castrop formula	Mean	0.2197	0.2074	0.3555	0.1989	0.1876	0.3218
	Standard deviation	0.0483	0.0418	0.0887	0.0494	0.0410	0.0958
	Median	0.2038	0.1945	0.3249	0.1819	0.1751	0.2854
	2.5% quantile	0.1785	0.1677	0.2866	0.1579	0.1488	0.2570
	97.5% quantile	0.3680	0.3295	0.6399	0.3499	0.3070	0.6266
Haigis formula	Mean	0.1987	0.1875	0.3217	0.1766	0.1666	0.2859
	Standard deviation	0.0430	0.0375	0.0784	0.0441	0.0365	0.0858
	Median	0.1848	0.1759	0.2941	0.1615	0.1554	0.2532
	2.5% quantile	0.1596	0.1509	0.2562	0.1395	0.1315	0.2268
	97.5% quantile	0.3322	0.2973	0.5753	0.3124	0.2738	0.5623

*Note*: On the left side the arithmetic mean, median, and 90% quantiles of the defocus equivalent are shown for the case where the uncertainties of the biometric measures, the lens labelling, and the axis alignment error are all considered. The right side shows the corresponding results for the case where only the uncertainites of the biometric measures are considered. 2.5% and 97.5% quantile refers to the lower and upper boundary of the 95% confidence interval.

**FIGURE 2:**
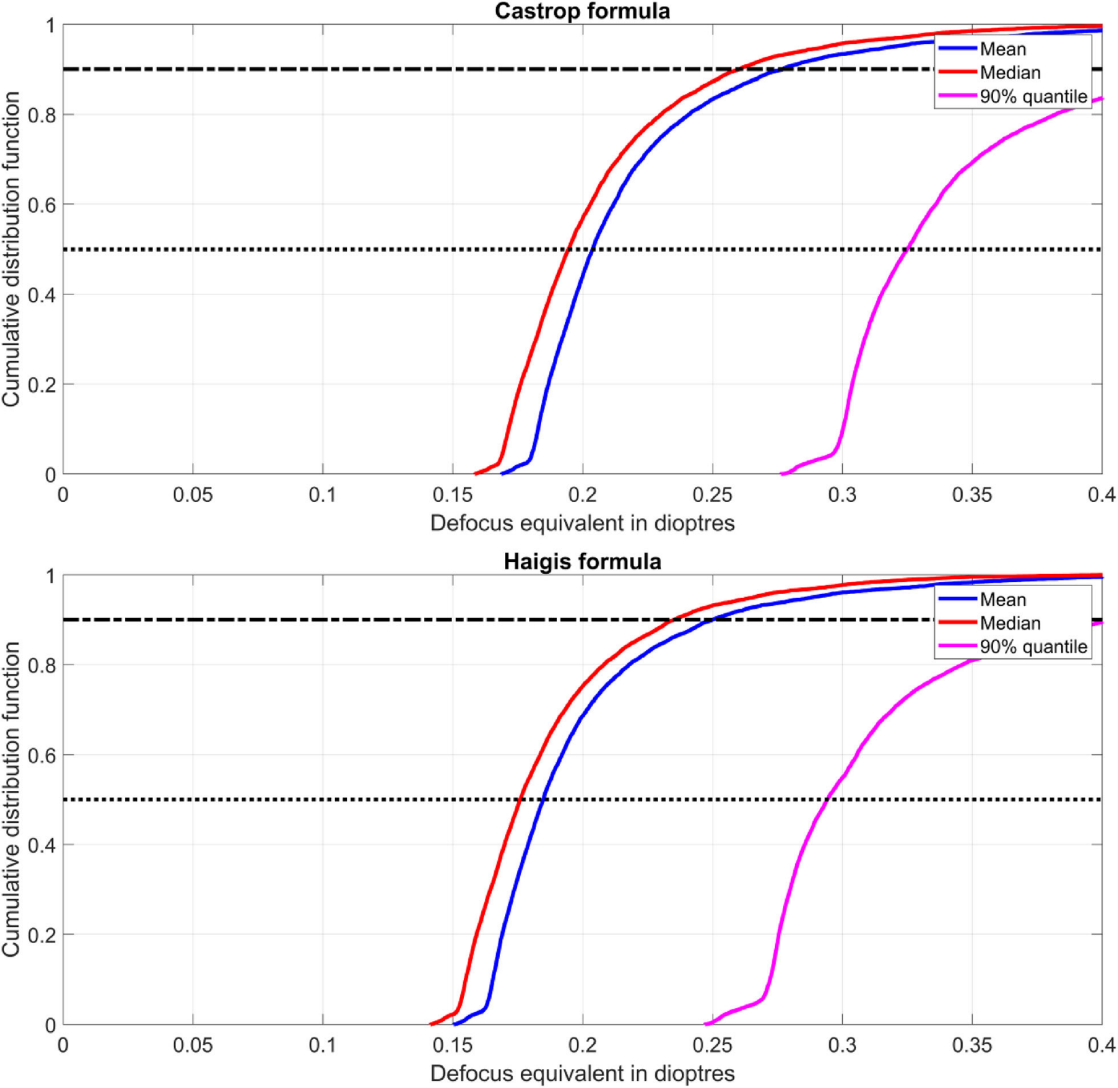
Cumulative distribution functions of the defocus equivalents as generated for each of the *n* = 7458 eyes in the study. In each case, the mean, median and 90% quantiles refer to the results for each eye as generated by the 100 000 Monte‐Carlo samples, and these are then plotted as a cumulative distribution with the vertical axis corresponding to the number of eyes. Values were derived with the Castrop formula as an example of a modern lens calculation concept based on a thick lens model for the cornea (upper graph), and the Haigis formula as an example of a classical 4th generation formula (lower graph). For this Monte‐Carlo simulation we considered variations of the biometric measures (axial length AL, anterior chamber depth ACD, central corneal thickness CCT, lens thickness LT, and keratometry with the corneal radius in the flat and steep meridian and the keratometric axis), the lens power labelling tolerances (truncated Gaussian distribution with standard deviation in equivalent and toric power as 1/3 of the permitted thresholds according to ISO 11979 and truncation according to ISO 11979), and the alignment error of the toric lens axis.

In Figure 2 the cumulative distribution function for the predicted mean, median and 90% quantile of the defocus equivalent is displayed, as derived with the Castrop formula (upper graph) and the Haigis formula (lower graph). From these graphs we can assess the weighting of the data (i.e. the percentage of DEQ results in each part of the range) and also easily determine the overall uncertainty of the predicted defocus equivalent for our study population.

**FIGURE 3:**
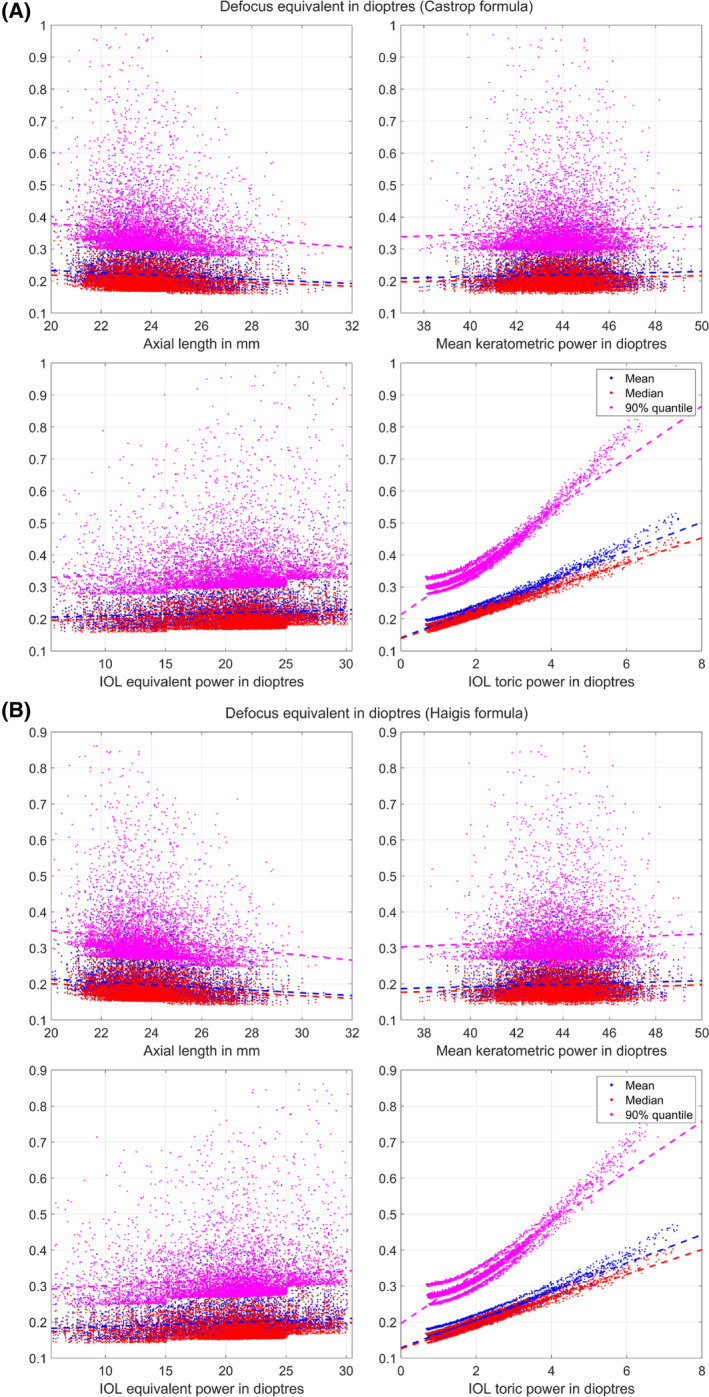
Predicted mean, median and 90% quantile of the defocus equivalent as a function of axial length (upper left plot), mean keratometric power (upper right plot), lens equivalent power (lower left plot), and lens toric power (lower right plot) derived with the Castrop formula as an example of a modern lens calculation concept based on a thick lens model for the cornea (A), and the Haigis formula as an example of a classical 4th generation formula (B). For this Monte‐Carlo simulation we considered variations of the biometric measures (axial length AL, anterior chamber depth ACD, central corneal thickness CCT, lens thickness LT, and keratometry with the corneal radius in the flat and steep meridian and the keratometric axis), the lens power labelling tolerances (truncated Gaussian distribution with standard deviation in equivalent and toric power as 1/3 of the permitted thresholds according to ISO 11979 and truncation according to ISO 11979), and the alignment error of the toric lens axis. All plots include the best fit trend line (dashed lines) which give some insight to the mean, median and 90% quantile defocus equivalent as a function of axial length, mean keratometric power, and the equivalent and toric power of the intraocular lens.

Figure 3 shows the trend of mean, median, and 90% quantile DEQ over the most relevant input parameters in the Monte‐Carlo simulation for the Castrop formula (Figure 3A) and the Haigis formula (Figure 3B) when the uncertainties in all input parameters are considered. The upper plots in both graphs indicate that predicted DEQ shows some decrease for long (myopic) eyes and some increase for steep corneas (with high mean keratometric power). The lower plots in both graphs show some increase in the predicted DEQ with larger equivalent lens power, and a strong increase with larger lens toric power. The increase of DEQ with larger equivalent and toric lens power is to be expected since the lens power labelling tolerances according to ISO 11979^14^ increase with the equivalent and the toric power. In addition, this effect is pronounced by the fact that with larger keratometric astigmatism the variation in keratometer axis Ax and with larger lens toric power the variation of the toric lens axis alignment magnifies the uncertainty of the refractive outcome.

Table 3 lists the median defocus equivalent DEQ for both the Castrop and Haigis formulae for Monte‐Carlo simulation runs in which only one predictor is varied at a time. The table shows that amongst the biometric measures the variations in axial distances (AL, ACD for the Castrop and the Haigis formula, and CCT and LT only for the Castrop formula) generally have a low impact on the predicted median DEQ, whereas the variations in keratometry (with the corneal radius in both meridians and the keratometric axis) dominates the median DEQ. Lens labelling tolerances (both in equivalent and toric power) appear to affect the DEQ by around 40%–50% of the effect of keratometry, and the alignment error of the toric lens by around 25%–30% of the effect of keratometry.

**TABLE 3:** Explorative data of the predicted median defocus equivalent at the spectacle plane resulting from a Monte‐Carlo simulation with 100 000 samples for each eye for variation of only one predictor.

		Variation only in								
Median DEQ in dioptres; *N* = 7458		AL	CCT	ACD	LT	Keratometry	Labelling tolerances	Lens axis alignment	Keratometry, labelling tolerances and lens axis alignment	|Keratometry and lens axis alignment
Castrop formula	Mean	0.0288	0.0009	0.0068	0.0062	0.1748	0.0760	0.0436	0.2039	0.1831
	Standard deviation	0.0038	0.0000	0.0017	0.0014	0.0356	0.0094	0.0263	0.0417	0.0411
	Median	0.0292	0.0009	0.0070	0.0064	0.1637	0.0748	0.0355	0.1909	0.1706
	2.5% quantile	0.0208	0.0008	0.0029	0.0032	0.1414	0.0590	0.0180	0.1645	0.1441
	97.5% quantile	0.0360	0.0010	0.0100	0.0089	0.2778	0.0987	0.1204	0.3258	0.3022
Haigis formula	Mean	0.0274	Not a predictor in the Haigis formula	0.0023	Not a predictor in the Haigis formula	0.1530	0.0740	0.0435	0.1843	0.1624
	Standard deviation	0.0033		0.0006		0.0305	0.0098	0.0260	0.0374	0.0365
	Median	0.0277		0.0023		0.1434	0.0731	0.0356	0.1728	0.1512
	2.5% quantile	0.0205		0.0009		0.1241	0.05559	0.0181	0.1484	0.1273
	97.5% quantile	0.0337		0.0034		0.2416	0.0972	0.1199	0.2940	0.2697

*Note*: AL refers to variation of axial length only, CCT to variation of central corneal thickness, ACD to variation of anterior chamber depth, LT to variation of lens thickness, Keratometry to variation of corneal radius in the flat and steep meridian and in the orientation of the flat axis, Labelling tolerances to variation in the labelled equivalent and toric power, and Lens axis alignment to the variation of the alignment of the lens orientation and the predicted lens target axis. In addition, the last two columns show the corresponding data taking keratometry, labelling tolerances, and lens axis alignment errors into account (penultimate column), or considering just variations in keratometry and lens axis alignment (final column). 2.5% and 97.5% quantile refers to the lower and upper boundary of the 95% confidence interval.

## 4 DISCUSSION

Although modern optical biometers show excellent performance and repeatability compared to the classical ultrasound or first generation optical biometers, there is still some measurement noise in the distance measurements (such as AL, CCT, ACD or LT) and in the results of keratometry (R1_a_, R2_a_ and Ax).^8^, ^9^, ^10^, ^11^ In biometers that provide data for the corneal back surface curvature in addition to keratometry, there is also some variability for repeat measurements of this curvature. However, the biometric parameters are only one potential source for prediction error in the refraction.^1^, ^5^, ^7^, ^16^ We also have to take into account the labelling tolerances of the lens.^4^, ^12^, ^13^, ^15^ In stigmatic lenses only the labelling tolerances of the (equivalent) power has to be considered, but in toric lenses we also have to consider the labelling tolerances of the toric power and the potential alignment error of the toric lens in the eye which may deviate from the target axis. Specifically, in the case of toric lenses the measurement noise is not restricted to the mean corneal radius or keratometric power: both the corneal curvature in the flat and the steep meridian as well as the keratometer axis could also show some variation.^8^, ^9^, ^10^, ^11^

The data in the literature about repeatability or reproducibility of biometers are neither consistent nor completely satisfactory: even when classical metrics such as the intra session standard deviation are properly reported, for an error propagation model the crosstalk between the parameter uncertainties is required.^10^ As an example, we are confident that there are some correlations between the measurement noise of all the distances in the eye. Specifically for the keratometry (or corneal back surface measurement) we expect that the measurement noise of the flat and steep meridians will be correlated to some extent and that the measurement noise in the keratometric axis will depend strictly on the amount of astigmatism. Even though classical error propagation concepts (for instance, Gaussian error propagation) do not benefit from this additional information, a Monte‐Carlo simulation would benefit significantly from the availability of this additional information.^1^, ^10^, ^12^, ^13^, ^23^

In the present study we used literature data reporting the repeatability metrics of a modern optical biometer^9^, ^11^ together with a large clinical dataset of eyes measured prior to cataract surgery to predict the refraction uncertainty after cataract surgery with implantation of a toric intraocular lens. To ensure a realistic simulation we restricted our dataset to eyes with some corneal astigmatism where a toric lens implantation is appropriate. In addition we restricted the parameter space to eyes which matched the delivery range of a modern toric lens model. These conditions could easily be adapted to any toric lens model with any delivery range and quantisation in the equivalent and toric power. In the absence of correlation information on the input data we assumed that noise in all of the biometric parameters, the lens power labelling tolerances (spherical equivalent and toric power) and in the alignment error of the toric lens axis are uncorrelated. Based on our dataset and the literature data on biometric uncertainties^9^, ^11^ and the lens power labelling tolerances according to ISO 11979^14^ we set up a Monte‐Carlo simulation to calculate the appropriate toric lens for each eye (*N* = 7458 toric lens power calculations). We then varied all of the input parameters according to a white noise profile (NMC = 100 000 variations) and back‐calculated the spherocylindrical refraction for each of the NMC·*N* = 745.8 millions of situations to obtain the defocus equivalent. This resulting DEQ was taken as a single metric to qualify the refractive outcome after cataract surgery with toric lens implantation. We feel that the defocus equivalent in this situation is appropriate since it refers to the length of the 3D power vector of the refraction prediction error. Because the defocus equivalent is always positive, the mean value alone might not fully describe the DEQ distribution. We therefore decided to provide the median and the 90% quantile as additional metrics to describe the DEQ.

The mean, median and 90% quantile statistics for our dataset considering all biometric uncertainties, lens power labelling tolerances and toric lens axis alignment errors are listed on the left side of Table 2. To separate the effect of noise in the biometric measurements from the variation in the lens power and axis alignment we performed a second Monte‐Carlo simulation on the same dataset where we zeroed all variations in the lens power labelling and the variations in the toric lens axis as shown on the right side of the table. The table shows that the predicted DEQ is slightly smaller when the uncertainties of the lens power labels and toric lens axis alignment are disregarded. In addition we see from the table that DEQ predicted using the Castrop formula is slightly larger as compared to the predicted DEQ from the Haigis formula. This is mostly due to the fact that the Castrop formula considers two more input parameters, and as all of these parameters show some (uncorrelated) noise the variation in the DEQ will be larger. However, this does not necessarily mean that the predicted DEQ will be larger with the Castrop formula than with the Haigis formula when used in a real life scenario, since we know that the formula prediction error itself will systematically decrease when including more (reliable) predictors in the lens power calculation strategy. In any case, a consideration of the formula prediction error is outside the scope of this study. This task would require reliable postoperative (spherocylindrical) refraction data for the entire dataset together with the equivalent and toric lens power and the (measured postoperative) orientation of the lens.

Figure 2 displays the cumulative distribution functions for the mean, median and 90% quantile defocus equivalent over the 100 000 Monte‐Carlo variations for our study population. To aid interpretation, and using the median defocus equivalent as an example, the graphs show that 50% of the study population have median defocus equivalent values below approximately 0.17 or 0.19 dioptres for the Haigis and Castrop formulae respectively (dotted horizontal line), and in 90% of the study population (dash‐dotted line), the median defocus equivalent is expected to be below 0.23 dioptres (for the Haigis formula) and below 0.26 dioptres for the Castrop formula. In contrast to the symmetrical ‘S' shaped plot that would be expected from a normal distribution, the plots in Figure 2 show a rapid initial rise, indicating a distribution skewed towards the lower values (i.e. that the bulk of the results are in the lower part of the DEQ range).

In Figure 3 we monitor the trend of the predicted DEQ as functions of the individual main predictors axial length, mean keratometric power, and equivalent and toric power of the lens. Comparing the results of the Castrop and Haigis formulae there are no systematic differences. If we consider a constant measurement noise over the entire parameter range then it is clear that the relative error in AL is lower in long eyes and therefore the predicted DEQ will decrease for long eyes. The positive trend of DEQ with mean keratometric power is not so readily apparent, as we used literature data with a constant measurement uncertainty for the flat and steep meridian over the entire range of keratometric power.^8^, ^9^, ^11^ However, since there is generally an inverse correlation between AL and keratometric power, DEQ shows some slight increase with the mean keratometric power. From the lower left plots we see that the DEQ systematically increases with the lens equivalent power. This is mostly a result of the labelling tolerances which increase stepwise at thresholds 15, 25 and 30 D. Since the lens model used for the simulation is only available in a range between 6 and 30 D we only see the steps at 15 and 25 D in the plot. Perhaps the most surprising result is that DEQ shows a tremendous increase with the toric lens power as shown in the lower right plots. However, this increase can easily be explained as an overlay of several effects: first of all, a large lens toricity is used with a large corneal astigmatism, and since we used a constant uncertainty of the keratometric axis over the entire range of keratometric power (and therefore keratometric astigmatism) the axis deviation affects the residual refraction much more in eyes with large corneal astigmatism than those with low corneal astigmatism. Secondly, as with the lens equivalent power, the labelling tolerances for the lens toric power increase with the toricity with the consequence that the variation in the postoperative refraction will be higher in lenses with large toricity. And last but not least, since we have assumed a constant alignment error distribution for the axis of the toric lens over the entire range of toricity, lenses with a larger lens torus will cause larger postoperative refraction errors (mostly cylindrical errors). This means that upgrading our Monte‐Carlo simulation would require not only detailed information about correlations of parameter uncertainties, but also some insight into the heteroscedasticity in order to determine whether the uncertainties show any variation over the entire parameter range. For example, considering noise in the keratometer axis we might expect it to decrease with increasing amount of keratometric astigmatism.

Our study has some limitations: For the biometric measures we considered the literature data on repeatability in terms of intrasession standard deviation Sw. However, we did not find data about correlations between parameter uncertainties in these papers, and therefore we assumed uncorrelated white noise for all parameters which might be a simplification, especially for the three elements of keratometry. Next, we did not find any data in the literature on heteroscedasticity of the uncertainties except for the power labelling tolerances where the tolerances are defined in the ISO 11979. Especially for the keratometric axis we would expect the repeatability to be better for larger corneal astigmatism values. And last but not least this Monte‐Carlo simulation considers only the variation of input parameters on the predicted refraction in terms of defocus equivalent. We did not include the formula prediction error itself, which could contribute to the predicted DEQ shown in this paper. Analysis of the formula prediction error would require reliable data on the refractive outcome as well as the equivalent and toric power of the implanted lens and a reliable postoperative measurement of the toric lens orientation, and is therefore beyond the scope of this paper.

In conclusion, our data show that a large portion of the defocus equivalent error after cataract surgery with toric lens implantation is explained by the uncertainty of the biometric measures, the lens power labelling tolerances (for equivalent and toric power), and the alignment error of the toric lens axis. Since the lens power labelling tolerances make a significant contribution to this portion of the overall DEQ, additional labelling of the exact equivalent and toric power on the lens package could help to improve postoperative results. To improve the Monte‐Carlo simulation additional studies to investigate the interaction of the parameter uncertainties as well as the heteroscedasticity or variation of the uncertainties over the entire parameter ranges would be required. In contrast to classical Gaussian error propagation Monte‐Carlo simulation could consider these additional data to obtain more realistic predictions of the refraction error resulting from input parameter variations.

## CONFLICT OF INTEREST STATEMENT

Langenbucher reports speaker fees from Hoya Surgical and Johnson & Johnson Vision outside the submitted work. Szentmáry and Cayless report no financial or proprietary interests. Cooke reports speaker fees from Alcon, Johnson & Johnson Vision and Heidelberg Engineering outside of the submitted work. Hoffmann reports speaker fees from Hoya Surgical and Johnson & Johnson outside the submitted work. Wendelstein reports research grants from Carl Zeiss Meditec AG, speaker fees from Carl Zeiss Meditec AG, Alcon, Rayner, Bausch and Lomb, and Johnson & Johnson Vision outside of the submitted work.

## Data Availability

The data that support the findings of this study are available from the corresponding author upon reasonable request.
